# Replacement of water yam (*Dioscorea alata* L.) indigenous root endophytes and rhizosphere bacterial communities *via* inoculation with a synthetic bacterial community of dominant nitrogen-fixing bacteria

**DOI:** 10.3389/fmicb.2023.1060239

**Published:** 2023-02-06

**Authors:** Sumetee Liswadiratanakul, Kosuke Yamamoto, Minenosuke Matsutani, Vatanee Wattanadatsaree, Shunta Kihara, Yuh Shiwa, Hironobu Shiwachi

**Affiliations:** ^1^Department of International Agricultural Development, Faculty of International Agriculture and Food Studies, Tokyo University of Agriculture, Tokyo, Japan; ^2^Department of Molecular Microbiology, Faculty of Life Sciences, Tokyo University of Agriculture, Tokyo, Japan; ^3^NODAI Genome Research Center, Tokyo University of Agriculture, Tokyo, Japan

**Keywords:** synthetic bacterial community, bacterial inoculation, plant growth-promoting bacteria, bacterial diversity, 16S rRNA, metagenomics, *Dioscorea alata* L., nitrogen-fixing bacteria

## Abstract

Biofertilizers containing high-density plant growth-promoting bacteria are gaining interest as a sustainable solution to environmental problems caused by eutrophication. However, owing to the limitations of current investigative techniques, the selected microorganisms are not always preferred by the host plant, preventing recruitment into the native microbiota or failing to induce plant growth-promoting effects. To address this, five nitrogen-fixing bacteria previously isolated from water yam (*Dioscorea alata* L.) plants and showing dominant abundance of 1% or more in the water yam microbiota were selected for analysis of their plant growth-promoting activities when used as a synthetic bacterial inoculant. Water yam cv. A-19 plants were inoculated twice at 10 and 12 weeks after planting under greenhouse conditions. Bacterial communities in root, rhizosphere, and bulk soil samples were characterized using high-throughput 16S rRNA amplicon sequencing. Compared with non-inoculated plants, all bacterial communities were significantly altered by inoculation, mainly at the genus level. The inoculation effects were apparently found in the root communities at 16 weeks after planting, with all inoculated genera showing dominance (in the top 35 genera) compared with the control samples. However, no significant differences in any of the growth parameters or nitrogen contents were observed between treatments. At 20 weeks after planting, the dominance of *Stenotrophomonas* in the inoculated roots decreased, indicating a decline in the inoculation effects. Interestingly, only the *Allorhizobium-Neorhizobium-Pararhizobium-Rhizobium* clade was dominant (>1% relative abundance) across all samples, suggesting that bacteria related to this clade are essential core bacteria for water yam growth. This is the first report on addition of a synthetic nitrogen-fixing bacterial community in water yam plants showing that native bacterial communities can be replaced by a synthetic bacterial community, with declining in the effects of *Stenotrophomonas* on the modified communities several weeks after inoculation.

## 1. Introduction

Biofertilizers (also known as bioinoculants, biocontrol agents, and biopesticides) are gaining interest as a sustainable solution to environmental problems caused by the inappropriate use of chemical compounds. Biofertilizers usually contain high-density living cells of different types of microorganism (e.g., bacteria or fungi), which colonize the rhizosphere or plant interior when applied to the seed, plant surface or soil (Mohammadi and Sohrabi, 2012; Berg et al., 2020). The most widely used microorganisms are plant growth-promoting bacteria (PGPB), which promote plant performance directly and/or indirectly *via* three primary modes of action: increased nutrient accessibility, production of plant growth regulator, and increased plant host systemic resistant induction/antagonistic activity (Mohammadi and Sohrabi, 2012; Expósito et al., 2015; Sathya et al., 2017). In addition, among all PGPB, nitrogen-fixing bacteria (NFB) are one of the most used microorganisms since nitrogen is one of the major limiting nutrients for plant growth and yield (Franche et al., 2009; Mohammadi and Sohrabi, 2012). Moreover, about 80% of the atmosphere is composed of gaseous nitrogen, but plants cannot directly use this form of nitrogen. It must first be converted through the process of biological nitrogen fixation by NFB (Mohammadi and Sohrabi, 2012). The NFB do not provide only fixed nitrogen to host plant, but they can also promote plant growth through other activities such as phosphate solubilization and indole-3-acetic acid production as well as siderophore production (Park et al., 2005; Rangjaroen et al., 2015).

The effects of PGPB have been documented not only in legumes plant but also in non-legume plants such as rice (Araújo et al., 2013; Rangjaroen et al., 2015), maize (Naveed et al., 2013; dos Santos et al., 2020), sweet potato (Farzana and Radizah, 2005; Dawwam et al., 2013), sugarcane (dos Santos et al., 2020), and vegetables (Flores-Félix et al., 2013; Chinakwe et al., 2019). However, previous studies on bacterial inoculation seem to face similar problems in terms of biofertilizer development, with many studies reporting significant plant growth-promoting (PGP) effects under *in vitro* conditions (e.g., growth chamber, sterile conditions) and others reporting deleterious effects or much fewer effects on plant growth and yield under field conditions (Valverde et al., 2007; Araújo et al., 2013; Botta et al., 2013; Naveed et al., 2013; Parnell et al., 2016; Hashmi et al., 2019). These conflicting results therefore suggest a gap between *in vitro* trials and effectiveness in the field in terms of efficiency (Boukhatem et al., 2022).

Previous studies suggest that the PGP effects of biofertilizers are dependent on the growth stage, environment, crop species, and cultivar (Hoflich, 2000; Zhang et al., 2011). Moreover, it has also been suggested that the beneficial effects observed under *in vitro* conditions are due to minimization or exclusion of competition between native microbes and unfavorable environmental conditions (Berg et al., 2020). It was also revealed that, in general, PGPB populations undergo a rapid decline soon after soil inoculation (Bashan et al., 2014) due to unfavorable abiotic conditions such as low pH, desiccation, salinity, and temperature (Singleton et al., 2002). Competition between single-strain inoculated bacteria and native microorganisms is also considered an important factor limiting effectiveness, with native microorganisms adapting quicker and showing greater compatibility with the host plants (Bashan et al., 2014). To overcome this, co-inoculation or inoculation with a bacterial consortium have been suggested, with the synergetic effects found to increase adaptation and result in more efficient plant growth compared with single-strain inoculants (Botta et al., 2013; Hashmi et al., 2019).

Compatibility between the host plant and introduced bacteria can also have differing or even deleterious PGP effects in different crop cultivars (Naveed et al., 2013). In this regard, plants are thought to possess different mechanisms aimed at the recruitment of specific microbes based on their genetics, growth stage, and environment, as well as in response to nutrient availability and stress (Hoflich, 2000; Zhang et al., 2011; Parnell et al., 2016). Additionally, Trabelsi and Mhamdi (2013) suggested that it is possible for host plants to replace their native PGPB with the introduced bacteria, which carry out the same or similar growth-promoting functions. It was also suggested that the effects of microbial inoculation on native microbial communities could last for several weeks before the community composition returns to its original state (Baudoin et al., 2009; Qiao et al., 2017; Thokchom et al., 2017). The colonization of introduced bacteria can be influenced by competition with other native endophytes for limited nutrients and niches. The competition can occur through root colonization (e.g., biofilm formation), production of antibiotic compounds that directly affect other microbe growth, or depletion of resources essential for other microbes (Berg et al., 2020; Santos et al., 2022). However, owing to the limitations of current investigative techniques and high analytical costs, studies into plant microbiota and the interaction between bacterial inoculates, the plant host, and microbial communities remain limited (Trabelsi and Mhamdi, 2013). Moreover, the selected inoculates are not always preferred by the host plant, preventing recruitment into the plant microbiota or failing to induce PGP effects.

Yams (*Dioscorea* spp.), annual tuberous crops, are an important staple for millions of people in tropical and subtropical regions (Raphael et al., 2015). Yams also serve as a major source of production in West Africa (Fu et al., 2011; Verter and Bečvářová, 2015). However, yam cultivation requires high levels of chemical fertilization to maintain productivity, resulting in high production costs (Carsky et al., 2010). Previous studies have reported the beneficial effects of inoculated bacteria on yam plants. The study of Jimtha et al. (2017) reported that tuber size and root number of *Dioscorea nipponica* plants were improved after inoculation with *Proteus* spp. R6. A recent study suggested that the individual inoculation of *Azotobacter chroococcum* DBC12 or *Azotobacter vinelandii* DBC9 with 50% of the recommended nitrogen fertilization level on *Dioscorea rotundata* Poir. showed highest yields compared with other treatments (Sanchez et al., 2021). Okigbo (2005) revealed the inoculation of antagonistic *Bacillus subtilis* significantly reduced or eliminated rot caused by *Aspergillus niger*, *Botryodiplodia theobromae,* or *Penicillium oxalicum* on yam tubers. Our previous studies by Takada et al. (2017, 2018) revealed comparable growth of water yam cv. A-19 (control) plants under low-fertility soil conditions compared with nitrogen fertilization treatment. They further revealed that 38.4% of nitrogen in the control plants was derived from the atmosphere. The authors suggest that these amounts of nitrogen were contributed by NFB. Subsequently, Liswadiratanakul (2020) also reported significant increases in most growth parameters and nitrogen content of water yam (*D. alata* L.) cv. A-19 inoculated with root endophytic NFB *Agrobacterium* sp. strain 343 under growth chamber conditions compared with the non-inoculated plants. It suggests the potential of inoculated NFB on yam production. The experimental design was subsequently repeated by Liswadiratanakul (2020) under greenhouse conditions. However, all growth parameters and the nitrogen content were not significantly different between treatments except for higher tuber dry weights observed in inoculated plants.

Therefore, to further address the difficulties associated with biofertilizer development, five NFB originating from water yam roots were selected based on their dominance (>1% relative abundance) in the water yam microbiota revealed by Kihara et al. (2022) and their PGP activities. The PGP effects of a synthetic bacterial inoculant on plant growth and native water yam microbiota were subsequently examined. To the best of our knowledge, this is the first report to describe the effects of inoculation of water yam with a synthetic bacterial community consisting of five dominant endophytic NFB, bringing new insight into the development of biofertilizers.

## 2. Materials and methods

### 2.1. Experimental design and plant material

This study was conducted in a greenhouse at Tokyo NODAI, Setagaya campus, Tokyo, Japan, from May 2020 to February 2021. Water yam cv. A-19 maintained by Tokyo NODAI was used in the pot test. Tuber rots (including wet rot, soft rot, and brown dry rot) caused by *Fusarium oxysporum*, *F. solani*, *Rhizopus nodosus*, and *Botryodiplodia theobromae* are the most serious pathogen for yam tuber during cultivation or storage. Reddy (2015) recommended the use of benomyl or thiabendazole to control 80–90% of rotting in the field. Therefore, thirty (30) whole yam tubers weighing approximately 60 g each were washed with tap water to remove the soil and surface sterilized in 10% (w/v) benomyl fungicide solution (Kumiai Chemical Co., Japan) for 10 min.

The tubers were cultivated (on May 13) in Wagner pots (size: 1/5000 a; Kiya Seisakusho Ltd., Tokyo, Japan) filled with 2.5 kg of sieved poor-nutrient soil (size less than 4.75 mm). Soil chemical properties were as follows: pH 5.8; electrical conductivity (EC) at 25°C, 119.1 mS m^−1^; cation exchange capacity (CEC), 11.3 meq 100 g soil^−1^; total N, 0.1 (%); total C, 1.8 (%); C/N ratio, 22.0; available P_2_O_5_, 7.4 mg 100 g soil^−1^; and total K, 0.5 mg g soil^−1^. The pots were placed in the greenhouse and maintained at an average temperature of 26.3°C. The experiment followed a completely randomized design with six repetitions per treatment. Plants were irrigated as required, and no fertilizer or pesticide was applied.

### 2.2. Bacterial inoculant and inoculation procedure

Previous studies have used microbial abundance to identify key species in the microbiota which high abundant taxa are considered as the core species (Müller et al., 2016; Schmitz et al., 2022). Therefore, we defined bacteria showing an abundance of 1% or more in the water yam microbiota as dominant and used these criteria to select dominant NFB in our culturable bacteria library derived from yam plants based on bacterial 16S rRNA metagenomic sequencing data reported by Kihara et al. (2022) (Supplementary Table 1 and Supplementary Figure 1). Seventeen (17) strains, previously isolated by Ouyabe et al. (2019), Takada (2020), and Liswadiratanakul et al. (2021), were selected (Table 1), all of which were related to the genera *Neorhizobium* and *Rhizobium*, *Enterobacter*, *Stenotrophomonas*, and *Ralstonia* at a relative abundance in the water yam plants of 21.16, 3.96, 12.53, and 2.10%, respectively. Next, we analyzed the *in vitro* PGP traits of 17 isolated bacterial strains to select one strain for each genus, which has higher PGP activities (Table 1; Ouyabe et al., 2019; Takada, 2020; Liswadiratanakul et al., 2021). Finally, S-60 (*Neorhizobium*), S-64 (*Rhizobium*), S-163 (*Enterobacter*), S-392 (*Stenotrophomonas*), and 1615 (*Ralstonia*) were combined for the synthetic bacterial community.

**Table 1:** Analysis of the *in vitro* plant growth-promoting traits of the isolated bacterial strains.

Strain accession	Closely related species	Yam accession	N_2_ fixation	PSI	IAA (μg/mL)
S-8	*Enterobacter roggenkampii*	A-18	+	1.7 ± 0.4	77.0 ± 6.4
S-12	*Rhizobium radiobacter*	A-18	+	1.3 ± 0.1	29.5 ± 0.2
S-17	*Rhizobium mesosinicum*	A-18	+	2.6 ± 0.1	0.6 ± 0.3
S-60	*Neorhizobium alkalisoli*	A-62	+	1.7 ± 0.1	3.2 ± 0.3
S-64	*Rhizobium tropici*	A-62	+	1.3 ± 0.1	57.7 ± 2.4
S-84	*Rhizobium tropici*	A-133	+	1.9 ± 0.1	2.1 ± 0.8
S-131	*Rhizobium capsici*	E-2	+	1.5 ± 0.0	5.9 ± 3.4
S-163	*Enterobacter bugandensis*	A-62	+	1.8 ± 0.0	87.2 ± 13.8
S-187	*Enterobacter huaxiensis*	E-3	+	1.8 ± 0.1	68.6 ± 0.9
S-324	*Rhizobium mesoamericanum*	A-19	+	1.2 ± 0.0	2.5 ± 1.6
S-346	*Rhizobium pakistanense*	A-62	+	NG	2.0 ± 0.8
S-350	*Rhizobium mesoamericanum*	A-62	+	1.5 ± 0.0	5.7 ± 2.9
S-363	*Rhizobium miluonense*	A-133	+	1.4 ± 0.1	3.8 ± 1.6
S-392	*Stenotrophomonas pavanii*	A-133	+	NG	10.6 ± 0.8
3E-17	*Rhizobium sp.*	A-19	+	2.1 ± 0.2	4.1 ± 0.9
1615	*Ralstonia sp.*	A-19	+	1.7 ± 0.2	35.6 ± 8.8
1620	*Rhizobium sp.*	A-19	+	NG	10.8 ± 0.4

N_2_ fixation ability of bacterium was previously described in Ouyabe et al. (2019), Takada (2020), and Liswadiratanakul et al. (2021). PSI, phosphate solubilization index; IAA, indole-3-acetic acid production; NG, negative result. Yam accession numbers were given by Tokyo University of Agriculture.

The processing of synthetic bacteria inoculation was performed by the modified method of Vafadar et al. (2013) and the brief processing is shown in Supplementary Figure 1. The selected bacteria were cultured separately for 24 h, inoculated on fresh LB liquid medium, and incubated at 28°C with shaking at 180 rpm. The concentrations of each bacterium were measured using a hemocytometer (Bacteria Counter A161, Sunlead Glass Co., Ltd.). The bacteria cultures were suspended together in fresh liquid LB medium at a final concentration of 10^+6^ cells / mL, respectively, then used to inoculate the water yam plants at 10 and 12 weeks after planting (WAP; on 24 July and 7 August, respectively). On each inoculation date, plants were inoculated on the top of soil with 100 mL bacterial inoculant or 100 mL cell-free LB liquid medium as a non-inoculated control (T0).

The indole-3-acetic acid (IAA) production was determined using a colorimetric method according to Gordon and Weber (1951). Briefly, 100 μL of 1-day-old bacterial suspension was transferred to a 50-mL flask containing 30 mL Luria Bertani (LB) liquid medium supplemented with L-tryptophan (0.5 mg mL^−1^). This was carried out in triplicate. The flasks were incubated at room temperature for 72 h on a rotary shaker. The resulting broth was centrifuged at 10,000 × *g* for 15 min at room temperature. A 500-μL sample of cell-free supernatant was collected and mixed with 1 mL of Salkowski reagent (1 mL of 0.5 M ferric chloride solution added to 50 mL of 35% of perchloric acid [HClO_4_]) and one drop of ortho-phosphoric acid (H_3_PO_4_). The samples were incubated at 30°C for 30 min before determining the absorbance at 530 nm using a spectrophotometer (U-2800, Hitachi High-Technologies Corporation) against an IAA standard solution with a concentration ranging from 5 to 50 μg mL^−1^. The ability of the selected strains to solubilize calcium phosphate [Ca_3_(PO_4_)_2_] was evaluated using Pikovskaya’s agar (Paul and Sinha, 2017) purchased from Hi-Media Laboratory Pvt. Ltd. (Mumbai, India). Solid Pikovskaya’s agar was prepared by dissolving 31.3 g in 1 L of ultra-pure water according to the manufacturer’s instructions. Bacteria were spotted on the medium using sterile toothpicks in triplicate and incubated at 30°C. The presence of visible clear halos around the colonies was observed after 7 days.

### 2.3. Plant growth parameters

Plants were destructively collected to evaluate the effects of inoculation on growth and the nitrogen content at 8 WAP (pre-inoculation; 56 days old; on July 8), 16 WAP (112 days old; September 4), and 20 WAP (140 days old; October 2). Plant growth was measured as leaf area, leaf, stem, and root dry weights, and the number and dry weight of new tubers. The leaf chlorophyll content was determined *via* SPAD (Soil Plant Analysis Development) values of three mature leaves taken from mid-sections of each plant, with three measurements per leaf, using a chlorophyll gauge (Konica Minolta Co. Japan.). On each sampling date, six fresh plants were randomly selected from each treatment and dried at 80°C to a constant dry weight. Dried samples were ground and sieved using a 0.5-mm mesh for further analysis.

N contents and N uptake in the leaf, stem, root, and tuber samples at each sampling point were determined using a nitrogen-carbon-hydrogen analyzer (SUMIGRAPH NCH-22F, Sumika Chemical Analysis Service, Ltd.). In the analyses, 10 mg of sieved leaf and 20 mg of all other sieved samples were used. Hippuric acid (N: 10.36% and C: 71.09%) was used as an internal standard and for calibration.

### 2.4. Sample preparation for metagenomic analysis

Fractionation of the roots, rhizosphere, and bulk soil was performed as previously described in Schlaeppi et al. (2014) at 8 WAP (pre-inoculation; 56 days old), 16 WAP (112 days old), and 20 WAP (140 days old). Briefly, three plants from each treatment were uprooted from their pots and remaining soil was removed by gently shaking without breaking the roots. Roots 0.5 to 5.5 cm long from the base of the seed tuber were placed in a 50-mL sterile plastic tube filled with 40 mL 1× phosphate-buffered saline containing 130 mM NaCl, 7 mM Na_2_HPO_4_, 3 mM NaH_2_PO_4_ (pH 7.0), and Silwet L-77 0.02% (v/v). The roots were washed by shaking at 180 rpm for 20 min then transferred to new 50-mL sterile plastic tubes and centrifuged at 4,000 × *g* for 20 min at room temperature. The supernatant was discarded and the remaining pellets were collected as rhizosphere samples (Rh). Root samples were obtained as described above followed by surface sterilization as follows: soaking in 70% ethanol for 2 min and 1% sodium hypochlorite (NaClO) for 5 min, respectively, then rinsing in sterile distilled water five times. Excess water on the roots was removed using sterile paper towels, then the roots were transferred to new 50-mL sterile plastic tubes as root samples (R). Soil samples were collected from non-planted pots and placed in 50-mL sterile plastic tubes as bulk soil samples (BK). All samples were frozen in liquid nitrogen for storage at −40°C until further use.

### 2.5. DNA extraction, PCR amplification, and construction of a gene clone library

R, Rh, and BK samples (0.25 g each) were used for microbial DNA extraction. R samples were frozen in liquid nitrogen then ground into a fine powder with a sterile mortar and pestle. Microbial genomic DNA was extracted using a DNeasy^®^ PowerSoil^®^ Kit (QIAGEN, Germany) according to the manufacturer’s instructions. The concentration of extraction products was improved by evaporating (EYELA CVE-2000, Tokyo Rikakikai co, Ltd). Next, 10 μL of 1 mM Tris–HCl (pH 8.5) was added to the evaporated samples then the DNA concentration was determined using a Qubit™ dsDNA HS Assay Kit (Thermo Fisher Scientific Inc., Waltham, MA, United States). DNA concentrations were adjusted to 5 ng μL^−1^ before preparation of a 16S Metagenomic Sequencing Library according to the Illumina 16S Sample Preparation Guide (15044223; Illumina Inc.).

Amplicon PCR was carried out at the hypervariable V5-V7 regions of the bacterial 16S rRNA using the following primers: forward illumine_799F, 5′-TCGTCGGCAGCGTCAGGATTAGATACCCKG-3′, and reverse illumine_1193R, 5′-GTCTCGTGGGCTCGGTCATCCCCACCTTCC-3′. Thermocycling was slightly modified to 95°C for 3 min, then 30 cycles of 95°C for 30 s, 55°C for 30 s, 72°C for 30 s, and 72°C for 5 min. The PCR products were subsequently amplified with dual indices and an Illumina sequencing adapter using a Nextera^®^ XT Index Kit V2 set D (Illumina Inc.). Next, 5 μL of R and 15 μL of Rh and BK DNA products were used as the DNA template for Index PCR, which was carried out as follows: 95°C for 3 min, followed by 16 cycles of 95°C for 30 s, 55°C for 30 s, 72°C for 30 s, and 72°C for 5 min.

PCR cleanup was then performed to purify the obtained products using AM Pure XP beads (Beckman Coulter Co, Ltd., Tokyo, Japan). The quality of each library was evaluated using an Agilent 2200 TapeStation (Agilent Technologies, Inc., Santa Clara, CA, United States) before diluting to a final concentration of 5 nmol L^−1^. Diluted 3-μL samples were then pooled together and sequenced as paired-end, 300-bp reads on an Illumina MiSeq sequencing platform (Illumina Inc.). Sequencing was performed twice, the first run with all samples and the second run to re-sequence those showing a non-saturated rarefaction curve. All sequenced data obtained in this study were deposited in the DDBJ Sequence Read Archive (DRA) database under accession number DRA014856.

### 2.6. Sequence processing and analysis

Sequence processing was performed as described by Zabat et al. (2018). Raw paired-end FASTQ files were quality filtered, trimmed, de-noised, and merged using DADA2 (Callahan et al., 2016) in QIIME 2 (ver. 2017.11). Chimeric sequences were identified and removed using the consensus method in DADA2. Using DADA2, sequences were clustered into amplicon sequence variants (ASVs) at 100% identity. Taxonomic analysis of the ASVs was performed using the QIIME 2 q2-feature-classifier plugin with a pre-trained Naïve Bayes classifier on the SILVA 99% ASV database (version 128) trimmed to the V5-V7 region of the 16S rRNA gene (DeSantis et al., 2006). Multiple alignment and phylogenetic reconstruction were carried out using MAFFT and FastTree, respectively (Price et al., 2009; Katoh and Standley, 2013).

### 2.7. Statistical analysis

Statistical analyses were performed with IBM Statistical Package for the Social Sciences (SPSS) Statistics 22 (IBM SPSS Statistics for Windows, Version 22.0. Armonk, NY, IBM Corp.). Means of each growth parameter and the N content were compared between treatments using the T-test (*p* = 0.05). Statistical analysis and visualization of the ASVs data were performed using QIIME 2 (Bolyen et al., 2019) and R software version 4.1.1 (R Core Team, 2021) using packages of iNEXT (Hsieh et al., 2016), tidyverse (Wickham et al., 2019), dunn.test (Dinno, 2017), and vegan (Oksanen et al., 2020). Alpha-diversity was performed based on sample-size-based rarefaction and coverage-based rarefaction (Chao and Jost, 2012) using the iNEXT package (Hsieh et al., 2016), with 50 bootstrap replicates per sample. The rarefaction curve was not plateaued at the same sample size for all samples. Species richness and Shannon indices based on the Effective Number of Species (ENS; Chao et al., 2014) were assessed using coverage-based rarefaction at a coverage of 98.5%, which corresponds to the lower coverage calculated for a sample using the iNEXT package (Hsieh et al., 2016).

The Kruskal-Wallis test, followed by a *post-hoc* Dunn test with Benjamin-Hochberg correction in R (Dinno, 2017), was performed to determine the effects of combined bacterial inoculation on the alpha-diversity indices of each sample type. The relationship between the bacterial structure and each sample type was visualized using Principal Coordinate Analysis (PCoA) based on weighted UniFrac distance matrix calculated from the raw ASV abundance table (non-rarefied) using permutational analysis of variance (PERMANOVA; Adonis function; 999 permutations) on QIIME2. The average relative abundance (*n* = 3) of each taxon was also statistically analyzed using a nonparametric Mann–Whitney test (*p* = 0.05) with IBM SPSS Statistics 22.

## 3. Results

### 3.1. PGP activities of the isolated bacterial strains

A total of 17 bacterial strains previously isolated by Ouyabe et al. (2019), Takada (2020), and Liswadiratanakul et al. (2021) were evaluated for their PGP activities through analysis of IAA production and P solubilization traits. The results are shown in Table 1. All bacteria showed positive response in terms of both traits, except for strains S-346, S-392, and 1,620, which were negative in the P solubilization assay. The best candidates in each genus were subsequently selected according to their PGP activities and vigor as described in “Materials and Methods.” The strains S-60 (*Neorhizobium*), S-64 (*Rhizobium*), S-163 (*Enterobacter*), S-392 (*Stenotrophomonas*), and 1615 (*Ralstonia*) were selected as a synthetic bacterial community consisting of dominant endophytic NFB for use in the inoculation experiment.

### 3.2. Effect of inoculation on plant growth and the nitrogen content

The growth parameters and nitrogen contents of the water yam plants under greenhouse conditions are shown in Table 2. No significant differences were observed between the control (T0) and inoculation (T1) treatments in any of the growth parameters or the nitrogen content at all sampling dates.

**Table 2:** Effect of inoculation on growth and nitrogen content of water yam cv. A-19.

WAP	Treatment	Leaf area (cm^2^ plant^−1^)	SPAD (plant^−1^)	Leaf dry weight (g plant^−1^)	Stem dry weight (g plant^−1^)
8	Pre- inoculation	1174.4 ± 143.3	32.8 ± 3.2	2.8 ± 0.2	2.5 ± 0.4
16	T0	1933.1 ± 447.3^ns^	36.7 ± 3.9^ns^	6.5 ± 1.6^ns^	5.8 ± 1.3^ns^
	T1	2052.4 ± 146.6	39.7 ± 3.9	6.5 ± 0.8	6.1 ± 0.7
20	T0	2510.5 ± 475.3^ns^	37.8 ± 9.2^ns^	9.9 ± 1.6^ns^	7.5 ± 1.5^ns^
	T1	2645.3 ± 451.8	42.0 ± 5.9	10.5 ± 1.7	8.7 ± 1.2
WAP	Treatment	Root dry weight (g plant^−1^)	Tuber dry weight (plant^−1^)	Plant N content (%)	N uptake (mg plant^−1^)
8	Pre-inoculation	2.0 ± 0.3	–	1.8 ± 0.3	137.1 ± 42.7
16	T0	5.5 ± 1.4^ns^	–	1.5 ± 0.1^ns^	248.3 ± 53.0^ns^
	T1	6.6 ± 1.9	–	1.5 ± 0.2	285.8 ± 22.4
20	T0	7.2 ± 2.1^ns^	1.3 ± 0.8^ns^	1.3 ± 0.2^ns^	334.4 ± 24.0^ns^
	T1	6.3 ± 0.6	0.4 ± 0.2	1.4 ± 0.3	335.2 ± 57.8

Values represent means followed by the standard deviation. “ns” indicates no significant difference on the same sampling date according to the T test (*p* = 0.05). WAP, weeks after planning.

### 3.3. Analyses of 16S rRNA gene sequencing data

To determine the effect of inoculation with the selected synthetic bacterial community on the native water yam microbiota, 45 samples were analyzed at three sampling dates (8 WAP [pre-inoculation], 16 WAP, and 20 WAP, respectively). A total of 19,952,612 raw reads were obtained from the Illumina MiSeq sequencing platform, ranging from 902,342 to 158,503 per sample. After read-quality filtering and denoising, a total of 8,549,069 reads were obtained, ranging from 487,306 to 51,794 reads per sample. A total of 323,130 ASVs were extracted, ranging from 60,363 to 10 reads per sample (Supplementary Table 2).

**Figure 1:**
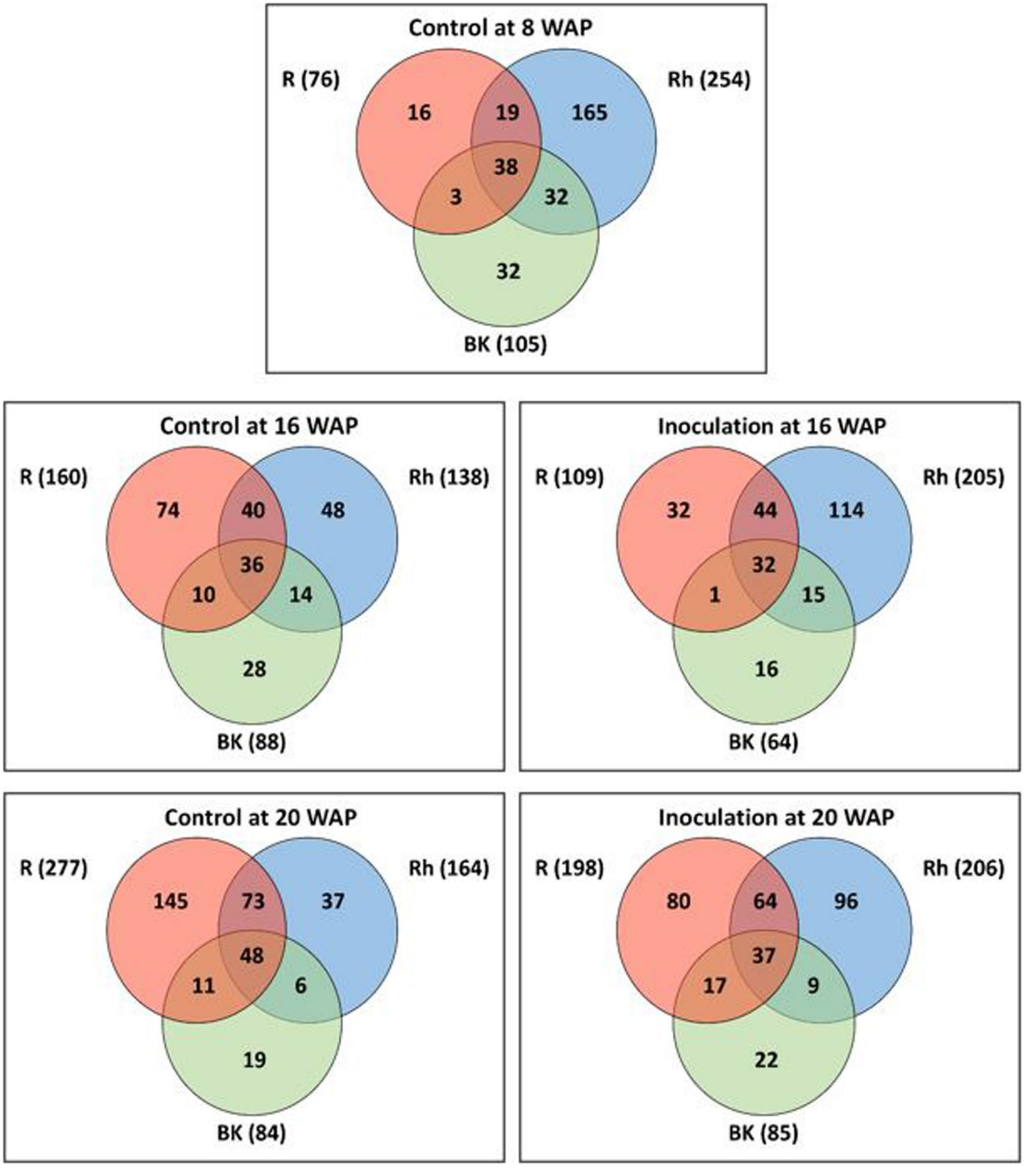
Venn diagrams showing the overlap between amplicon sequence variants (ASVs) under each treatment at 8 (pre-inoculation), 16, and 20 weeks after planting (WAP). Numbers in the brackets represent the total number of ASVs in the root (R), rhizosphere (Rh), and bulk soil (BK) samples under each treatment at each time point.

As shown in Supplementary Figure 2, the rarefaction curves did not plateau at the same sample size for all samples (Supplementary Figure 2A), and therefore, rarefaction curves were also obtained based on coverage-based rarefaction (Supplementary Figure 2B) to ensure that bacterial species from each sample were observed at the same level. Accordingly, the findings indicated that most of the bacterial species were sampled. Based on Venn diagram analysis (Figure 1), 76, 254, and 105 ASVs were identified before inoculation (8 WAP) in the R, Rh, and BK samples, respectively. A total of 38 ASVs were shared across the samples, while 16, 165, and 32 ASVs were unique to each sample type, respectively. At 16 WAP, the number of ASVs in the Rh (205) samples increased under inoculation treatment compared with the control (138), while the number in the R (109) and BK (64) samples decreased (160 and 88, respectively). Similarly, at 20 WAP, the inoculated Rh samples showed an increase in ASVs (206) compared with the control (164), while the inoculated R samples (198) showed a decrease compared with the control (277). Meanwhile, a similar number of ASVs were observed in the BK samples between inoculation and control treatments. At all dates and treatments, the R, Rh, and BK samples shared a similar number of ASVs (ranging from 32 to 48). Interestingly, only 27 taxa from 2,213 ASVs were common between the R and Rh samples at all harvesting dates and treatments, and these belonged to the phyla *Actinobacteria* (13), *Bacteroidetes* (1), *Firmicutes* (2), and *Proteobacteria* (11) at 48.1, 3.7, 7.4, and 40.7%, respectively (Supplementary Table 3).

### 3.4. Effect of inoculation on alpha and beta-diversities

**Figure 2:**
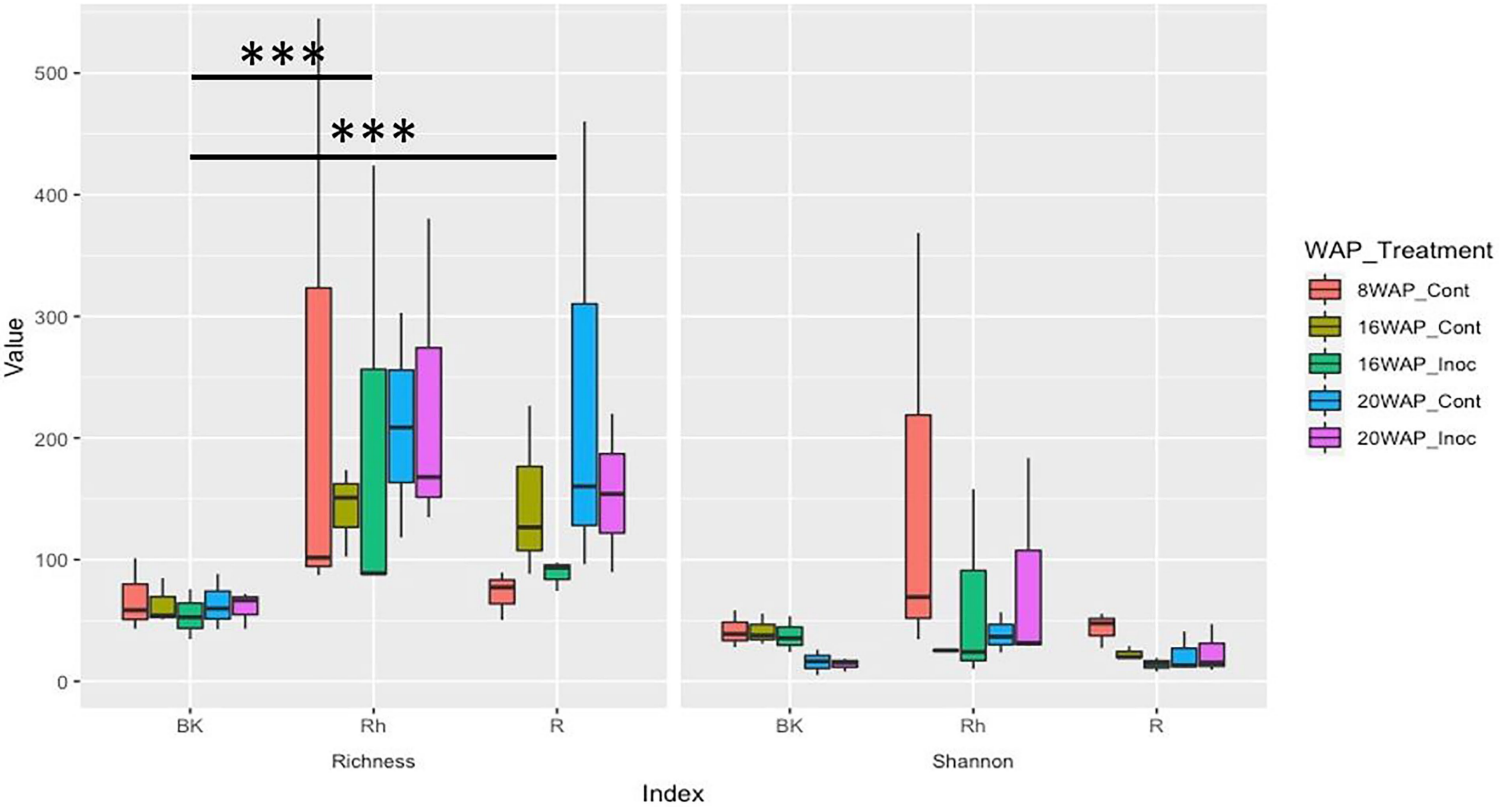
Alpha-diversity indices of the 16S rRNA gene sequences as determined by the species richness **(Left)** and Shannon indices **(Right)** based on the effective number of species using coverage-based rarefaction at a rate of coverage of 98.5% corresponding to the lower range in the iNext package of the R software. Cont, control non-inoculated treatment; Inoc, inoculation treatment; WAP, weeks after planting; R, root; Rh, rhizosphere; BK, bulk soil.

**Figure 3:**
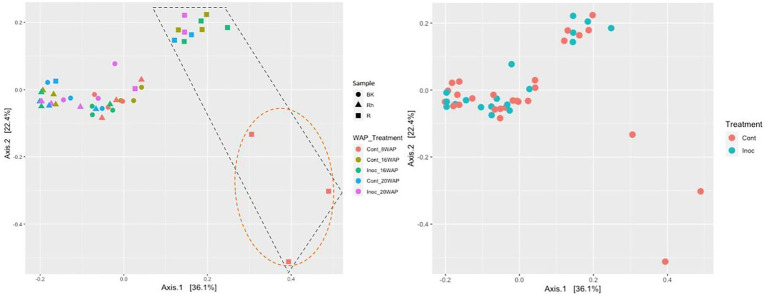
Principal coordinates analysis of the bacterial communities in each sample type **(Left)** and under each treatment **(Right)** generated based on weighted UniFrac distances using permutational analysis of variance (PERMANOVA; Adonis function; 999 permutations) on QIIME2. Cont, control non-inoculated treatment; Inoc, inoculation treatment; WAP, weeks after planting; R, root; Rh, rhizosphere; BK, bulk soil. The R bacterial structures were clustered separately from the other samples as shown in the black dashed box. At 8 WAP, the R bacterial structures were grouped separately from the other R samples as shown in the orange dashed circle.

The alpha-diversities of each sample type at each harvesting date were determined based on analysis of species richness and Shannon indices using ENS (Figure 2). The R and Rh samples showed highly significant species richness compared with the BK samples (*p* = 3.23E-04 and *p* = 5.91E-06, respectively), whereas no significant differences were observed within each sample type. The Shannon indexes exhibited no significant differences between or within each sample type. Beta-diversity analysis using principal coordinate analysis (PCoA) revealed that the bacterial structure of the R samples differed from that of the Rh and BK samples (36.1% of the variation), although the results were not affected by treatment or growth stage, except in the R samples at 8 WAP (Figure 3).

### 3.5. Effect of inoculation on the native bacterial communities at the phylum and class levels

**Figure 4:**
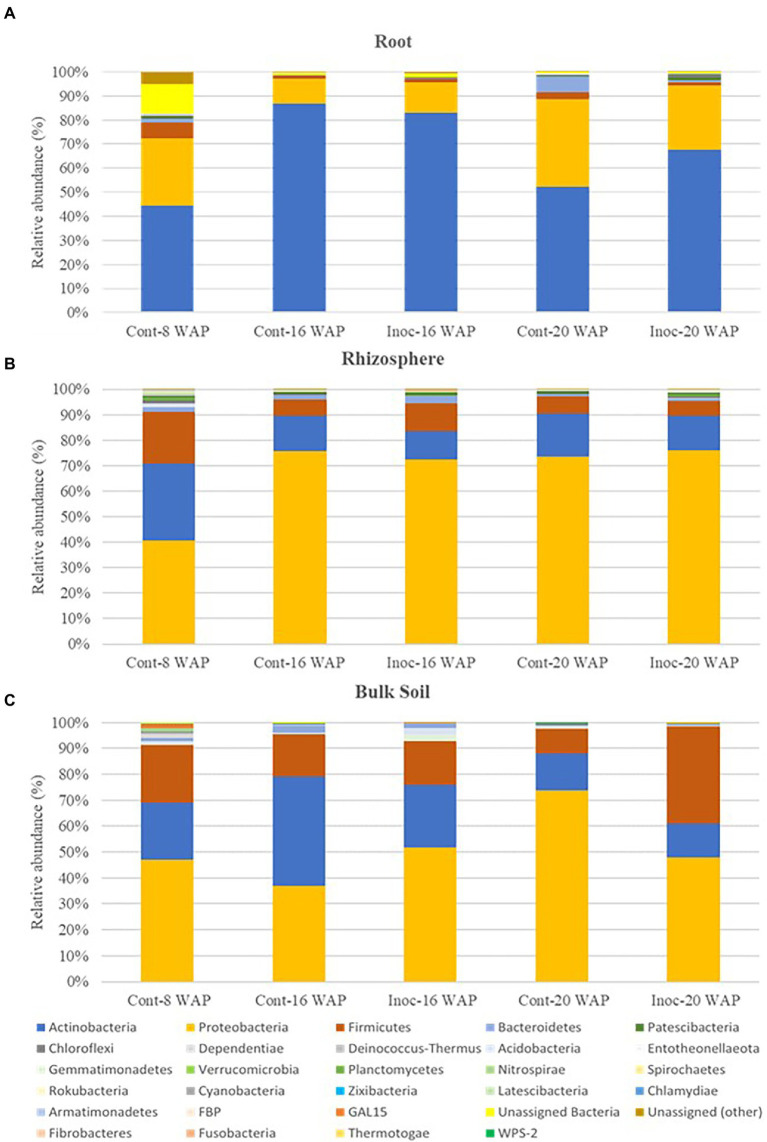
Average relative abundance of bacteria at the phyla level in the root **(A)**, rhizosphere **(B)**, and bulk soil bacterial communities **(C)**. Cont, control non-inoculated treatment; Inoc, inoculation treatment; WAP, weeks after planting.

Native bacterial communities of the water yam plants at the phylum and class level were apparently affected by inoculation with the synthetic bacterial community (Figure 4 and Supplementary Tables 4, 5). Before inoculation (8 WAP), across all sample types, the predominant bacteria (>10% of high-quality sequences) belonged to the phyla *Actinobacteria* (21.86–44.54%), *Proteobacteria* (27.79–47.03%), and *Firmicutes* (6.69–22.53%). At the class level, relative abundances were assigned to predominant classes *Alphaproteobacteria* (8.19–20.65%), *Gammaproteobacteria* (15.45–36.41%), *Actinobacteria* (20.28–44.18%), and *Bacilli* (6.28–21.77%) across all pre-inoculated samples. Predominant phyla in the BK bacterial communities were *Proteobacteria* (37.07–73.90%), *Actinobacteria* (13.19–42.12%), and *Firmicutes* (9.69–27.42%) across all BK samples at 16 and 20 WAP. At 16 WAP, the relative abundance of *Actinobacteria* and *Bacteroidetes* decreased from 42.12 to 23.86% and from 2.65 to 1.77%, respectively, compared with the control BK samples. Increases in *Proteobacteria* (from 37.07 to 51.93%), *Firmicutes* (from 16.22 to 17.10%), *Gemmatimonadetes* (from 0.44 to 2.42%), and *Acidobacteria* (from 0.28 to 2.37%) were observed. At 20 WAP, the inoculated BK samples showed decreases in phyla *Proteobacteria* (from 73.90 to 47.91%), *Actinobacteria* (from 14.04 to 13.19%), and *Acidobacteria* (from 1.08 to 0.09%) and an increase in *Firmicutes* (from 9.69 to 37.42%) compared with the control BK samples. However, significant differences were found only in *Actinobacteria* and *Proteobacteria* at 16 WAP, and *Acidobacteria* at 20 WAP (Supplementary Table 4).

As with the BK samples, the R bacterial communities were predominated by the phyla *Actinobacteria* (52.33–86.77%) and *Proteobacteria* (10.31–36.48%), and sub-dominated (>1% of high-quality sequences) by *Firmicutes* (1.43–2.68%) across all R samples at 16 and 20 WAP. Compared with the control samples, the relative abundance of *Actinobacteria* in the inoculated R samples decreased from 86.77 to 82.94%, while *Proteobacteria* and *Firmicutes* increased from 10.31 to 12.65% and from 1.43 to 1.69%, respectively, at 16 WAP. On the other hand, the abundances of *Proteobacteria*, *Firmicutes*, and *Bacteroidetes* in the inoculated R samples decreased from 36.48 to 26.41%, from 2.68 to 1.43%, and from 6.74 to 0.82%, respectively, at 20 WAP, while *Actinobacteria*, *Patescibacteria*, and *Chloroflexi* increased from 52.33 to 67.79%, from 0.15 to 1.20%, and from 0.38 to 1.41%, respectively.

The predominant phyla across all Rh samples were *Proteobacteria* (72.37–76.10%), *Actinobacteria* (11.06–16.77%), and *Firmicutes* (5.89–10.91%) at 16 and 20 WAP. At 16 WAP, a decrease in *Proteobacteria* (from 75.67 to 72.37%) and *Actinobacteria* (from 13.81 to 11.06%) and an increase in *Firmicutes* (from 6.34 to 10.91%) and *Bacteroidetes* (from 1.79 to 3.01%) were observed following inoculation. However, at 20 WAP, a decrease in *Actinobacteria* (from 16.77 to 13.36%) and *Firmicutes* (from 6.79 to 5.89%) and an increase in *Proteobacteria* (from 73.69 to 76.10%) was observed compared with the control.

Bacterial communities in the R, Rh, and BK samples at the class level are shown in Supplementary Table 5. At 16 WAP, the inoculated BK samples showed decreases in classes *Actinobacteria* (from 39.82 to 23.25%) and *Thermoleophilia* (from 1.72 to 0.61%), with increases in *Alphaproteobacteria* (from 13.81 to 18.05%) and *Gammaproteobacteria* (from 22.93 to 33.88%) compared with the control samples. Meanwhile, decreases in classes *Alphaproteobacteria* (from 8.22 to 5.03%) and *Gammaproteobacteria* (from 65.68 to 42.88%) were observed in the inoculated samples at 20 WAP, with an increase in class *Bacilli* (from 8.95 to 35.11%).

At 16 WAP, most sequences in the control R and Rh samples were assigned to classes *Actinobacteria* (86.63% in R) and *Alphaproteobacteria* (66.67% in Rh), while in both the inoculated R and Rh samples, decreases in the relative abundance of classes *Alphaproteobacteria* and *Actinobacteria* were observed (to 2.87 and 82.91% in R and to 25.67 and 10.28% in Rh, respectively). An increase in class *Gammaproteobacteria* was also observed in the R and Rh samples (from 4.50 to 9.75% and from 8.85 to 46.59%, respectively). Compared with the control samples, similar fluctuations were observed in the inoculated Rh samples at 20 WAP, with a decrease in the relative abundance of classes *Alphaproteobacteria* (from 50.40 to 47.65%) and *Actinobacteria* (from 16.55 to 12.60%) and an increase in *Gammaproteobacteria* (from 23.15 to 27.90%). On the other hand, decreases in classes *Alphaproteobacteria* (from 17.34 to 7.04%) and *Bacilli* (from 2.64 to 1.42%) were observed in the inoculated R samples, with an increase in *Actinobacteria* (from 52.01 to 67.76%). Moreover, at 16 WAP, significant differences in classes *Thermoleophilia* (R), *Alphaproteobacteria* and *Gammaproteobacteria* (Rh), and *Actinobacteria* (BK) were observed in the inoculated samples.

### 3.6. Effect of inoculation on the native bacterial communities at the family and genus levels

**Figure 5:**
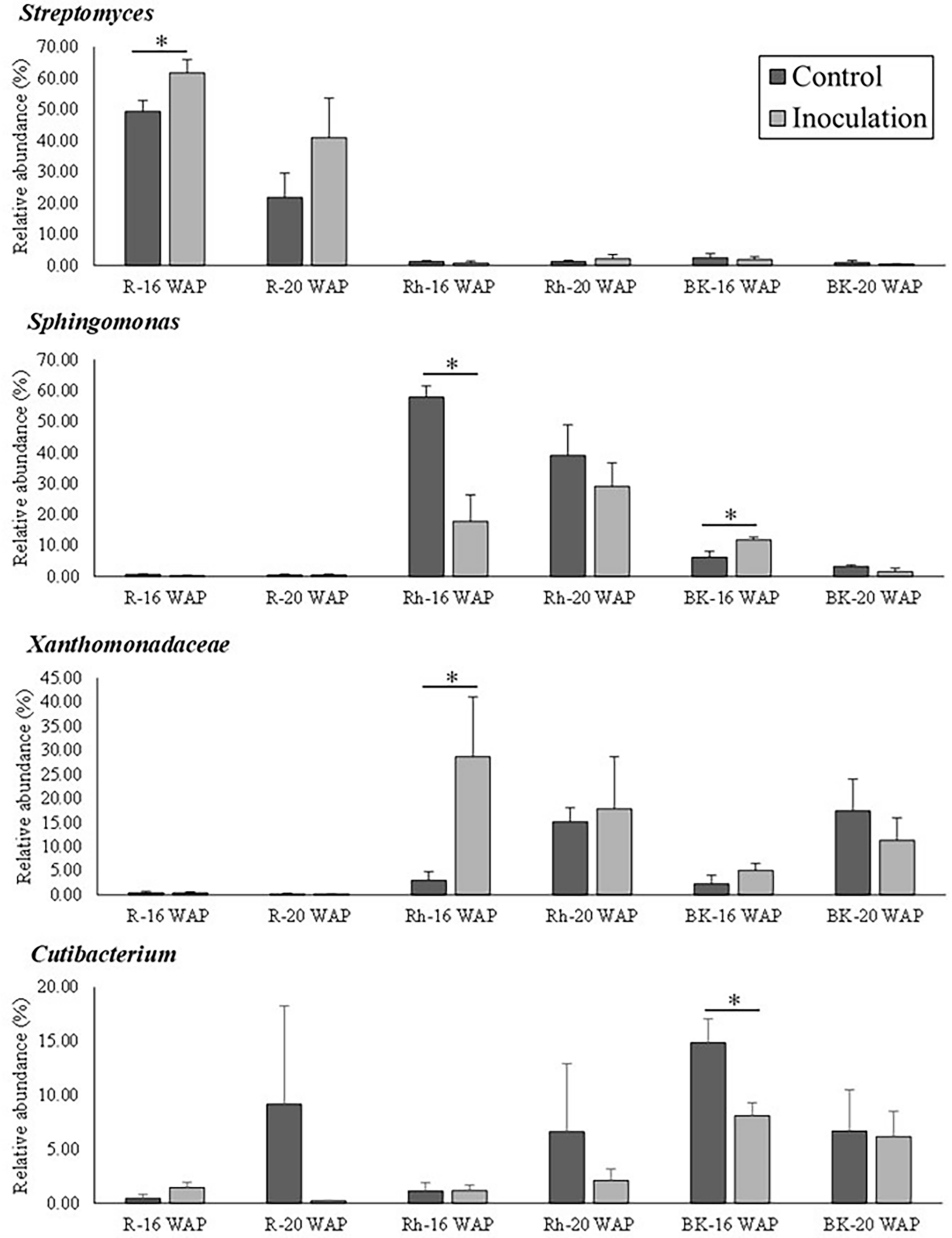
Relative abundance of bacteria at the genus/family level between control and inoculation treatment as affected by inoculation with the synthetic bacterial community. Asterisks indicate statistical significance (*p* = 0.05) according to a nonparametric Mann-Whitney test. WAP, weeks after planting; R, root; Rh, rhizosphere; BK, bulk soil.

The effect of inoculation on the native bacterial structure of the water yam plants was also characterized at the family and genus level. The top 35 dominant taxa at the family and genus level are shown in Supplementary Tables 6–8. Inoculation had a significant effect on the predominant taxa (>10% of high-quality sequences) as shown in Figure 5, with each sample type dominated by different predominant taxa, sub-dominant taxa (>1% of high-quality sequences), and rare taxa (<1% of high-quality sequences). Pre-inoculation (8 WAP), the R samples were mainly dominated by *Streptomyces* (18.20%), while *Staphylococcus* dominated the Rh and BK samples (16.05 and 15.06%, respectively). At 16 and 20 WAP, the BK bacterial communities were dominated by similar taxa across all samples (*Bacillus* [0.002–23.51%], *Cutibacterium* [6.16–14.87%], *Lysobacter* [4.41–38.67%], *Sphingomonas* [1.62–11.85%], and *Xanthomonadaceae* [2.26–17.45%]) (Supplementary Table 8). At 16 WAP, the inoculated BK samples showed decreases in *Cutibacterium* (from 14.87 to 8.11%), *Staphylococcus* (from 9.59 to 6.81%), and *Lawsonella* (from 4.75 to 1.76%) and increases in *Sphingomonas* (from 6.27 to 11.85%), *Lysobacter* (from 4.41 to 7.64%), and *Xanthomonadaceae* (from 2.26 to 5.08%) compared with the control samples. On the other hand, at 20 WAP, decreases in *Lysobacter* (from 38.67 to 22.94%), *Xanthomonadaceae* (from 17.45 to 11.35%), and *Sphingomonas* (from 3.35 to 1.62%) were observed, along with increases in *Bacillus* (from 0.0022 to 23.51%) and *Staphylococcus* (from 5.77 to 9.98%). However, only taxa related to *Cutibacterium* and *Sphingomonas* at 16 WAP and *Sphingomonadaceae* at 20 WAP showed significant differences between treatments (Figure 5 and Supplementary Table 8).

The effects of inoculation on the R bacterial communities are shown in Figure 5 and Supplementary Table 6. Compared with the control samples, the relative abundance of *Streptomyces* increased significantly at 16 WAP (from 49.34 to 61.61%), while that of *Nocardia* (from 10.13 to 8.61%), *Amycolatopsis* (from 7.75 to 5.59%), and *Glycomyces* (from 4.13 to 0.27%) decreased. Similarly, at 20 WAP, decreases in the abundance of the *Burkholderia-Caballeronia-Paraburkholderia* clade (from 9.20 to 5.73%), *Chryseobacterium* (from 6.17 to 0.35%), and *Cutibacterium* (from 9.17 to 0.23%) were observed compared with the control, with increases in *Streptomyces* (from 21.72 to 40.91%) and *Amycolatopsis* (from 11.19 to 17.89%).

At 16 and 20 WAP, the Rh bacterial communities across all Rh samples were dominated by *Sphingomonas* (17.78–57.89%) and *Xanthomonadaceae* (3.05–28.70%), and sub-dominated by the *Allorhizobium-Neorhizobium-Pararhizobium-Rhizobium* clade, *Bacillus*, *Cutibacterium*, *Lysobacter*, and *Staphylococcus* (Figure 5 and Supplementary Table 7). Compared with the control Rh samples, the inoculated samples showed a significant decrease in the relative abundance of *Sphingomonas* (from 57.89 to 17.78%), *Qipengyuania* (from 2.11 to 0.34%), and *Terrabacter* (from 1.76 to 0.30%) at 16 WAP, with increases in *Xanthomonadaceae* (from 3.05 to 28.70%) and *Pseudolabrys* (from 0.04 to 0.57%) (Figure 5 and Supplementary Table 7). Similarly, at 20 WAP, the relative abundance of *Xanthomonadaceae* increased (from 15.20 to 17.88%), while that of *Sphingomonas* decreased (from 39.09 to 29.11%) (Supplementary Table 7).

Compared with the control samples, inoculation affected taxa related to the introduced bacteria in the R communities at 16 WAP, with increases in the relative abundance of *Stenotrophomonas*, *Enterobacter*, and *Ralstonia* to 1.46, 1.04, and 0.21%, respectively, and decreases in *Rhizobium/Neorhizobium* to 2.04% at 16 WAP (Table 3). Even though only a small number of the introduced bacteria were altered, these bacteria dominated the top 35 bacteria within the R communities. Moreover, at 20 WAP, the relative abundance of *Enterobacter* increased significantly following inoculation (Table 3). The abundance of introduced bacteria also increased in the Rh samples at 16 WAP compared with the control, but only ASVs related to *Enterobacter* and *Rhizobium/Neorhizobium* were found in the top 35 dominant bacteria. However, fewer effects were observed in the BK bacterial communities. New genera were also observed following inoculation at 16 and 20 WAP, with the introduction of *Streptococcus* and *Anaerobacillus* in the R samples, *Neisseria* and *Capnocytophaga* in the Rh samples, and *Curvibacter* and *Nakamurella* in the BK samples.

**Table 3:** Relative abundance (percent of total good-quality sequences) of the introduced bacteria in the root (R), rhizosphere (Rh), and bulk soil (BK) samples as affected by inoculation with the synthetic bacterial community.

WAP	Treatment	R			
		*Rhizobium / Neorhizobium*	*Stenotrophomonas*	*Enterobacter*	*Ralstonia*
8	Pre-inoculation	8.25	0.00	0.00	0.00
16	T0	2.25	0.01	0.00	0.00
	T1	2.04	1.46	1.04	0.21
20	T0	15.37	0.02	0.04	0.00
	T1	3.15	0.03	3.25*	0.23
WAP	Treatment	Rh			
		*Rhizobium / Neorhizobium*	*Stenotrophomonas*	*Enterobacter*	*Ralstonia*
8	Pre-inoculation	3.42	0.00	0.00	0.00
16	T0	1.58	0.00	0.00	0.01
	T1	2.78	0.17*	0.43	0.25
20	T0	3.67	0.01	0.11	0.00
	T1	3.46	0.14	0.33	0.13
WAP	Treatment	BK			
		*Rhizobium / Neorhizobium*	*Stenotrophomonas*	*Enterobacter*	*Ralstonia*
8	Pre-inoculation	0.75	0.59	0.00	0.00
16	T0	1.10	0.58	0.00	0.46
	T1	0.66	0.00	0.00	0.00
20	T0	0.34	0.00	0.00	0.00
	T1	0.37	0.07	0.00	0.00

Values represent the average relative abundance. Asterisks (*) indicate a significant difference between treatments on the same harvesting date according to a nonparametric Mann–Whitney test (*p* = 0.05). Numbers in red indicate 0% relative abundance. WAP, weeks after planting.

## 4. Discussion

To further understand the effect of bacterial inoculation on water yam plants, we investigated the effects of inoculation with a synthetic bacterial community consisting of five dominant endophytic NFB, which were selected based on their dominance in water yam microbiota (>1% relative abundance) and their PGP activities, on plant growth and native bacterial communities. To the best of our knowledge, this is the first study to report the inoculation effects of a synthetic bacterial community on the growth and native bacterial communities of water yam plants.

Bacterial diversity and abundance in the water yam plants were evaluated using species richness and Shannon indices based on ENS and PCoA as alpha- and beta-diversities, respectively. Analysis of species richness revealed a significantly higher number of species (richness) in the R and Rh samples compared to the BK samples (Figure 2), inconsistent with the results of Kihara et al. (2022) who characterized the bacterial communities of water yam cv. A-19 under two different fertilization regimes (with- and without chemical fertilizers) using high-throughput 16 s rRNA amplicon sequencing. The highest richness and Shannon index values were observed in the BK samples, although no significant differences were observed between these and the R and Rh samples. Moreover, PCoA analysis revealed three sample-based clusters: BK, R, and Rh, and Leaf and Stem. In contrast, beta-diversity grouped the R samples separately (36.1% of the variation) to the Rh and BK samples in the present study (Figure 2). However, fertilization had no significant effect on bacterial structure in either experiment.

In this study, the dominant phyla in the R bacterial communities were *Actinobacteria* followed by *Proteobacteria* and *Firmicutes*, while Rh and BK were dominated by *Proteobacteria*, *Actinobacteria* then *Firmicutes* across all harvesting dates and treatments (Supplementary Table 4). The study of Palaniyandi et al. (2013) has well reviewed the beneficial effects of *Actinobacteria* associated with yam rhizosphere and their contribution to different PGP activities such as IAA production, tricalcium phosphate solubilization, and ACC deaminase activity as well as the control of foliar diseases in yam by producing antibiotics. However, the dominant phyla, in the present study, differ from those of Kihara et al. (2022), who revealed dominance of the phylum *Proteobacteria* followed by *Actinobacteria, Patescibacteria, Acidobacteria*, and *Firmicutes* across all samples and treatments. These differences in the bacterial communities of water yam cv. A-19 plants between studies are thought to be related to a number of factors, such as the identification technique, the environment, soil type, and plant growth stage (Zhang et al., 2011; Trabelsi and Mhamdi, 2013; Chaparro et al., 2014). In this study and that of Kihara et al. (2022), 16S sequencing was carried out using the same Illumina MiSeq benchtop sequencer, although the hypervariable regions of the bacterial 16S rRNA genes that were amplified differed. In this study, amplicon PCR was carried out at the hypervariable V5-V7 regions of the bacterial 16S rRNA, while Kihara et al. (2022) targeted the hypervariable V3-V4 regions. Moreover, in Kihara et al. (2022), the plants were grown in Shimajiri-maji subsoil on Miyako Island, Okinawa, Japan (Kihara et al., 2022), while in the present study, low-fertility Kanto-Loam soil from Tokyo was used. This was in line with the study of İnceoğlu et al. (2012) who characterized the rhizosphere communities of five different potato cultivars under two soil types using PCR denaturing gradient gel electrophoresis (PCR-DGGE). The author reported that the rhizosphere communities clearly differed between the two soils. Lundberg et al. (2012) also reported the pyrosequencing of the bacterial 16S rRNA gene of more than 600 *Arabidopsis thaliana* plants grown under controlled conditions in two natural soils. The results showed that root and rhizosphere communities are strongly influenced by soil type.

Also, this study found that root bacterial communities are affected by plant growth stage. Across all control R samples, the bacterial communities of the water yam plants were dominated by different groups depending on the growth stage (Supplementary Table 4), suggesting specific requirements of bacterial diversity and function at different stages of growth. These findings were in line with the study of Zhang et al. (2011) who suggested that plant growth development was the main factor affecting bacterial communities of soybean roots. The authors evaluated the effects of rhizobial inoculation (with *Bradyrhizobium liaoningense* CCBAU 05525), cropping systems (monoculture vs. intercropping with maize), and plant growth stage (vegetative, full-flowering, and seed-forming) on the diversity of soybean root endophytic bacteria using PCR-based terminal restriction fragment length polymorphism (T-RFLP) with 16S rRNA profiling. According to cluster analysis, the T-RFLP profiles showed that the bacterial communities of the soybean roots were mainly affected by growth stage followed by the cultivation system and rhizobial inoculation. The authors also suggested that soybean plants select different symbionts and endophytes at each growth stage using different mechanisms. However, growth stage was not thought to be the major factor affecting bacterial abundance since samples in both this study and that of Kihara et al. (2022) were collected within 120 days after planting.

This study also determined the effects of inoculation on the growth and N content of the water yam plants. Accordingly, no significant differences in any of the growth parameters or nitrogen contents were observed between the control (T0) and inoculation (T1) treatments at any of the sampling dates (Table 2). Trabelsi and Mhamdi (2013) previously suggested that host plants can replace their native PGPB with the introduced bacteria, which carry out the same or similar growth-promoting functions. They concluded that this mechanism may lead to no differences in growth between control and inoculated plants. This was confirmed in this study, whereby the R bacterial communities, which represented genera closely related to the introduced bacteria, became more dominant (in the top 35) in the inoculated R plants at 16 WAP (Supplementary Table 6), while growth remained similar between the control and inoculated plants.

Water yam cv. A-19 plants are thought to have a strong association with their beneficial native NFB. This has been supported by Takada et al. (2017, 2018), who revealed comparable growth of control water yam cv. A-19 plants under low-fertility soil conditions compared with nitrogen fertilization treatment. They further revealed that 38.4% of nitrogen in the control plants was derived from the atmosphere. Similarly, a recent study by Kihara et al. (2022) reported no significant effects of urea application on growth of water yam cv. A-19 plants compared to non-fertilized plants, with the native bacterial communities dominated by PGPB such as *Allorhizobium-Neorhizobium-Pararhizobium-Rhizobium* clade, *Burkholderia-Caballeronia-Paraburkholderia* clade, *Stenotrophomonas*, and *Pseudomonas* across all samples.

The strong association between water yam cv. A-19 plants and their native PGPB was also supported by the core bacteria identified in this study (Tables 4A, B and Supplementary Table 3). Of 2,213 ASVs, only 27 were found to be common between the R and Rh samples across all harvesting dates and treatments. Moreover, most promote plant growth through N_2_ fixation, P solubilization, and IAA production, or possess multiple plant growth-promoting abilities as shown in Tables 4A, B. However, the level of interaction between the plant host and native microorganisms is thought to vary, resulting in three levels of association: strong, moderate, and low. To further understand the degree of interaction in water yam, additional experiments are now needed with various water yam varieties differing in their growth abilities under low soil fertility.

**Table 4A:** The 27 taxa comprising core bacteria of the water yam (*Dioscorea alata* L.) bacterial microbiota and their possible plant growth-promoting functions.

Taxonomy	Possible functions	References
*Actinobacteria* (13)		
*Amycolatopsis*	IAA production, siderophore production, production of cellulase, lipase, protease, chitinase, hydrocyanic acid, and glucanase.	Alekhya and Gopalakrishnan (2016)
*Arthrobacter*	N_2_-fixation, IAA production, ACC deaminase synthesis, ammonia production, thiosulfate oxidation.	Siddikee et al. (2010)
*Corynebacterium 1*	N_2_-fixation, P solubilization, ACC deaminase synthesis, ammonia production, thiosulfate oxidation.	Siddikee et al. (2010), Sellstedt and Richau (2013), and Chinakwe et al. (2019)
*Cutibacterium*	Unknown.	–
*Dermacoccus*	P solubilization, IAA production, siderophore production.	Rangseekaew et al. (2021)
*Lawsonella*	Unknown.	–
*Lechevalieria*	Unknown.	–
*Leifsonia*	P solubilization, IAA production, Siderophore production, antifungal activity, ammonia production, Gibs production.	Kang et al. (2014)
*Micrococcaceae*	N_2_-fixation, IAA production, ACC deaminase synthesis, ammonia production, Gibs production.	Siddikee et al. (2010) and Boukhatem et al. (2022)
*Mycobacterium*	N_2_-fixation, P solubilization, IAA production, ACC deaminase synthesis.	Karmakar et al. (2021)
*Nocardia*	P solubilization, IAA production, Siderophore production, ammonia production, production of cellulase, amylase and protease.	Nimnoi et al. (2010) and Gong et al. (2018)
*Nocardioides*	N_2_-fixation, IAA production, ammonia production, induction of systemic-acquired resistance.	Conn et al. (2008) and Liotti et al. (2018)
*Streptomyces*	N_2_-fixation, P solubilization, IAA production, siderophore production, antifungal activity, secondary metabolite production.	Sellstedt and Richau (2013), Jog et al. (2014), and Olano et al. (2008)
*Bacteroidetes* (1)		
*Chryseobacterium*	N_2_-fixation, P solubilization, IAA production, siderophore production, ACC deaminase synthesis.	Mareque et al. (2015) and Youseif (2018)

N_2_, nitrogen; P, phosphate; IAA, indole-3-acetic acid; ACC, aminocyclopropane-1-carboxylic acid; Gibs, Gibberellins.

**Table 4B:** The 27 taxa comprising core bacteria of the water yam (*Dioscorea alata* L.) plant bacterial microbiota and their possible plant growth-promoting functions.

Taxonomy	Possible functions	References
*Firmicutes* (2)		
*Bacillus*	N_2_-fixation, P solubilization, IAA production, siderophore production, ACC deaminase synthesis.	de Melo Pereira et al. (2012) and Babu et al. (2013)
*Staphylococcus*	P solubilization, IAA production, ACC deaminase synthesis, improved salt and drought tolerance.	Shahid et al. (2019) and Jayakumar et al. (2020)
*Proteobacteria* (11)		
*Allorhizobium-Neorhizobium-Pararhizobium-Rhizobium*	N_2_-fixation, P solubilization, IAA production, siderophore production, ACC deaminase synthesis.	Duan et al. (2009) and Ouyabe et al. (2020)
*Bosea*	IAA production, siderophore production, ACC deaminase synthesis.	Bertola et al. (2019)
*Bradyrhizobium*	N_2_-fixation, ACC deaminase synthesis.	Terakado-Tonooka et al. (2013) and Ikeda et al. (2014)
*Burkholderiaceae*	N_2_-fixation, P solubilization, IAA production, siderophore production, cellulolytic enzyme production.	Rangjaroen et al. (2015)
*Burkholderia-Caballeronia-Paraburkholderia*	N_2_-fixation, P solubilization, IAA production, siderophore production, cellulolytic enzyme production.	Rangjaroen et al. (2015) and Kuramae et al. (2020)
*Enterobacteriaceae*	N_2_-fixation, P solubilization, IAA production, siderophore production, anti-microbial activity.	de Melo Pereira et al. (2012) and Naveed et al. (2013)
*Lysobacter*	N_2_-fixation, siderophore production, anti-microbial activity.	Iwata et al. (2010) and Expósito et al. (2015)
*Pseudomonas*	N_2_-fixation, P solubilization, IAA production, antifungal activity, ammonium production.	de Melo Pereira et al. (2012) and Ngoma et al. (2013)
*Ramlibacter*	Unknown.	–
*Sphingomonas*	N_2_-fixation, P solubilization, IAA production, siderophore production, cellulolytic enzyme production.	Rangjaroen et al. (2015)
*Xanthomonadaceae*	N_2_-fixation P solubilization, IAA production, antifungal activity, ammonium production.	Taulé et al. (2012) and Ngoma et al. (2013)

N_2_, nitrogen; P, phosphate; IAA, indole-3-acetic acid; ACC, aminocyclopropane-1-carboxylic acid; Gibs, Gibberellins.

In the present study, the inoculation effects of the synthetic bacterial community also had an effect on bacterial diversity of the R samples at 16 WAP whereby genera related to the introduced bacteria became the top 35 dominant bacteria in the R bacterial communities (Supplementary Table 6). Moreover, these effects of inoculation on the R bacterial communities were maintained at 20 WAP by remaining bacteria related to genera *Enterobacter* and *Ralstonia* in the top 35 dominant bacteria. However, the abundance of *Stenotrophomonas* at 20 WAP decreased compared with its abundance at 16 WAP, suggesting a decline in the inoculation effects. This finding is also in line with previous studies (Baudoin et al., 2009; Qiao et al., 2017), whereby microbial inoculation of the soil was found to alter the native microbial communities for several weeks, although the community composition often returned to its original state thereafter. Thokchom et al. (2017) also reported that the inoculation effects on the PGP rhizosphere under non-sterile natural soil conditions altered the microbial composition in the soil and roots for several months.

Moreover, in both experiments, only the *Allorhizobium-Neorhizobium-Pararhizobium-Rhizobium* clade was found in all sample types, with a relative abundance of >1% in the water yam microbiota at all sampling dates. In Kihara et al. (2022), the relative abundance of the *Allorhizobium-Neorhizobium-Pararhizobium-Rhizobium* clade accounted for 4.62–21.16% across the R, Rh, stem, and leaf samples, while in the present study, the R and Rh communities were also dominated by the *Allorhizobium-Neorhizobium-Pararhizobium-Rhizobium* clade (1.58–15.37%) across all harvesting dates and treatments. These results suggest that the *Allorhizobium-Neorhizobium-Pararhizobium-Rhizobium* clade comprises essential core bacteria even under different growth conditions.

Overall, this study is the first to report that the native bacterial communities of water yam cv. 19 can be replaced by a synthetic bacterial community, with the abundance of *Stenotrophomonas* decreasing several weeks after inoculation. Future studies are now required to determine the inoculation effects of synthetic bacterial communities consisting of essential core bacteria in the *Allorhizobium-Neorhizobium-Pararhizobium-Rhizobium* clade on the native bacterial communities and growth of various water yam varieties under different soil properties. Furthermore, it is necessary to include not only nitrogen-fixing bacteria, but also plant growth-promoting bacteria in the bacterial selection for which an *in vivo* assay is required to determine their PGP activities.

## Data availability statement

The data presented in the study are deposited in the DDBJ repository (https://www.ddbj.nig.ac.jp/), accession number DRA014856.

## Author contributions

SL, KY, and HS designed the experiment. SL and VW performed the experiments and measurements. SL, KY, MM, YS, and SK performed the data analyses. SL, KY, YS, and HS wrote the manuscript. All authors have read and agreed to the final version of the manuscript.

## Funding

This research was financially supported by the Japan Society for the Promotion of Science (KAKENHI; grant number: 18KT0046), Japan.

## Conflict of interest

The authors declare that the research was conducted in the absence of any commercial or financial relationships that could be construed as a potential conflict of interest.

## Publisher’s note

All claims expressed in this article are solely those of the authors and do not necessarily represent those of their affiliated organizations, or those of the publisher, the editors and the reviewers. Any product that may be evaluated in this article, or claim that may be made by its manufacturer, is not guaranteed or endorsed by the publisher.
