# Supplementary material for: Replacement of water yam (Dioscorea alata L.) indigenous root endophytes and rhizosphere bacterial communities via inoculation with a synthetic bacterial community of dominant nitrogen-fixing bacteria
Source: Front Microbiol. 2023 Feb 6;14:1060239. doi: 10.3389/fmicb.2023.1060239 (PMC9939703; doi:10.3389/fmicb.2023.1060239)
Supplement: Supplementary file 1 [file Image_1.pdf]

Criteria for selecting nitrogen-fixing bacteria (NFB)

1.) Having an abundance of 1% or more in the water yam microbiota

2.) Selecting NFB from our culturable bacteria library

3.) Having high plant growth-promoting activities (e.g., IAA production and P solubilization) and vigor

↓

Five (5) dominant endophytic NFB were selected as a synthetic bacterial community. (i.e., S-60 (*Neorhizobium*), S-64 (*Rhizobium*), S-163 (*Enterobacter*), S-392 (*Stenotrophomonas*), 1615 (*Ralstonia*)

↓ Culturing separately for 24 h in fresh LB liquid medium

Mix in liquid LB medium at a final concentration of 10^{+6} cells / ml, respectively

↓

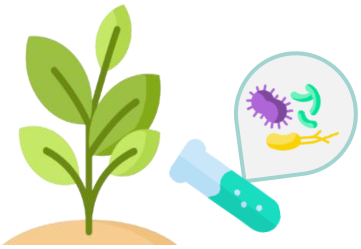

Inoculate water yam plants on the top of soil with 100 ml bacterial inoculant at 10 and 12 weeks after planting (WAP)

↓

Collecting plant samples at

- 8 WAP (Pre-inoculation; 56 days old)
- 16 WAP (112 days old)
- 20 WAP (140 days old)

- Estimation of growth parameters
- 16S metagenomic sequencing (Amplifying at V5-V7)

Supplementary Figure 1 Experimental workflow in this study. The images used in this figure were from Flaticon.com.
