# Supplementary material for: Replacement of water yam (Dioscorea alata L.) indigenous root endophytes and rhizosphere bacterial communities via inoculation with a synthetic bacterial community of dominant nitrogen-fixing bacteria
Source: Front Microbiol. 2023 Feb 6;14:1060239. doi: 10.3389/fmicb.2023.1060239 (PMC9939703; doi:10.3389/fmicb.2023.1060239)
Supplement: Supplementary file 2 [file Image_2.pdf]

A)

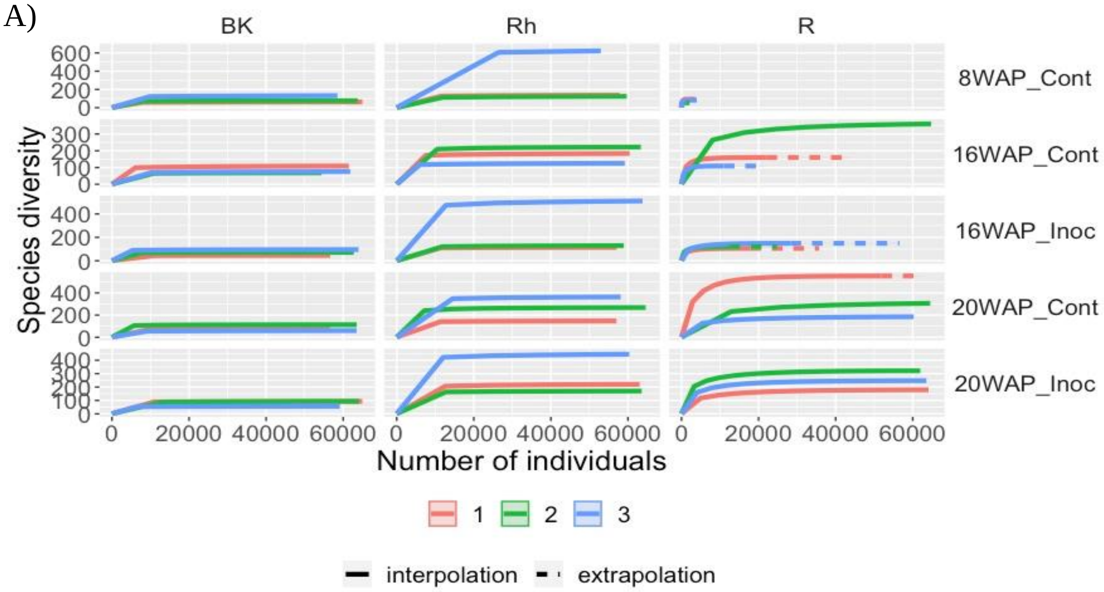

B)

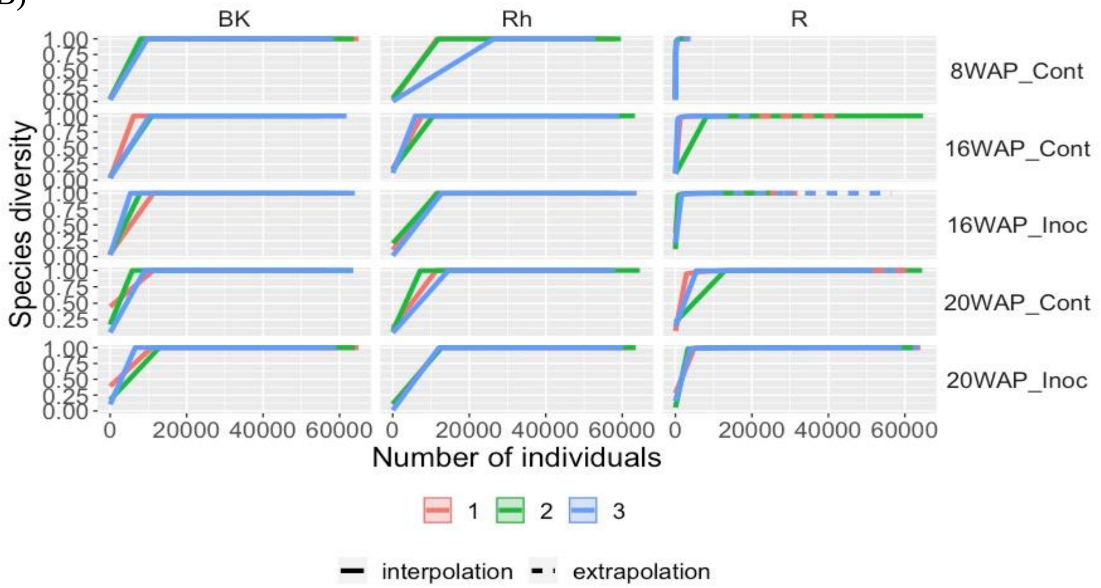

Supplementary Figure 2 Rarefaction curves for each treatment performed based on sample-size based rarefaction **(A)** and coverage-based rarefaction **(B)** at 8 (pre-inoculation), 16 and 20 weeks after planting (WAP). Curves were analyzed using the iNext package in R software, with 50 bootstrap replicates per sample. The three replicates per sample type (root [R], rhizosphere [Rh], and bulk soil [BK]) are shown in red, green, and blue, respectively.
