# Supplementary material for: Replacement of water yam (Dioscorea alata L.) indigenous root endophytes and rhizosphere bacterial communities via inoculation with a synthetic bacterial community of dominant nitrogen-fixing bacteria
Source: Front Microbiol. 2023 Feb 6;14:1060239. doi: 10.3389/fmicb.2023.1060239 (PMC9939703; doi:10.3389/fmicb.2023.1060239)
Supplement: Supplementary file 3 [file Image_3.pdf]

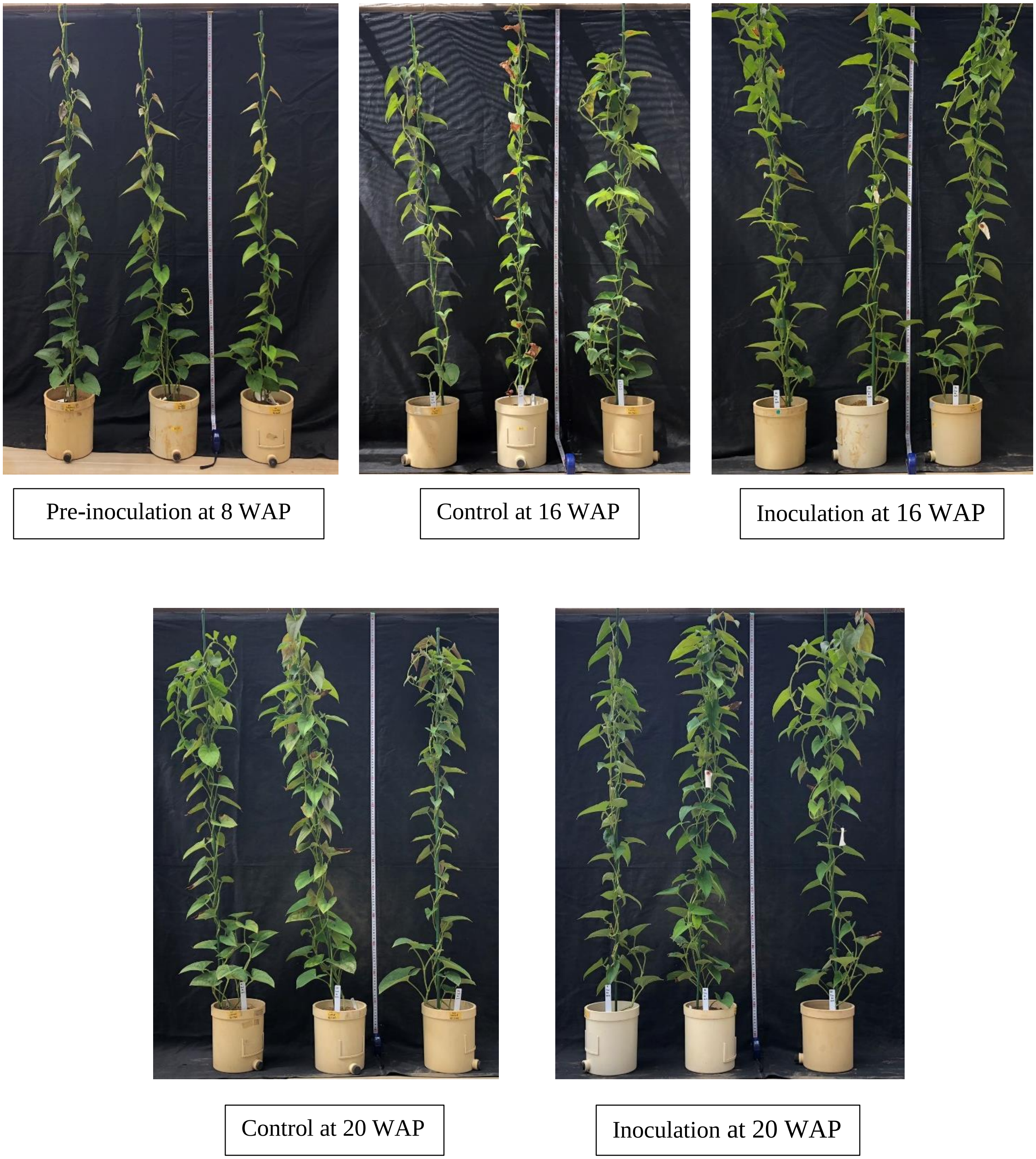

Supplementary Figure 3 Phenotypes of the water yam (*D. alata* L.) cv. A-19 plants 8 (pre-inoculation), 16, and 20 weeks after planting (WAP) as affected by inoculation with the synthetic bacterial community.
